# Decreasing Inappropriate Use of Antibiotics in Primary Care in Four Countries in South America—Cluster Randomized Controlled Trial

**DOI:** 10.3390/antibiotics6040038

**Published:** 2017-12-14

**Authors:** Inés Urbiztondo, Lars Bjerrum, Lidia Caballero, Miguel Angel Suarez, Monica Olinisky, Gloria Córdoba

**Affiliations:** 1 The Research Unit for General Practice and Section of General Practice, Department of Public Health, University of Copenhagen, 1353 Copenhagen, Denmark; inesurbiztondo@gmail.com (I.U.); lbjerrum@sund.ku.dk (L.B.); 2 Dr. Pedro Baliña Hospital, Public Health Ministry, Posadas 3300, Misiones, Argentina; lidia.gladis@gmail.com; 3 Policlínica Central de la Caja Nacional de Salud, La Paz 15000, Bolivia; sucumian@gmail.com; 4 Department of Family and Community Medicine, Faculty of Medicine, University of the Republic, Montevideo 11600, Uruguay; molinisky@gmail.com

**Keywords:** antibiotics, educational intervention, general practice

## Abstract

High antibiotic prescribing and antimicrobial resistance in patients attending primary care have been reported in South America. Very few interventions targeting general practitioners (GPs) to decrease inappropriate antibiotic prescribing have been investigated in this region. This study assessed the effectiveness of online feedback on reducing antibiotic prescribing in patients with suspected respiratory tract infections (RTIs) attending primary care. The aim was to reduce antibiotic prescribing in patients with acute bronchitis and acute otitis media. Both are RTIs for which antibiotics have a very limited effect. A cluster randomized two-arm control trial was implemented. Healthcare centres from Bolivia, Argentina, Paraguay and Uruguay participating in the quality improvement program HAPPY AUDIT were randomly allocated to either intervention or control group. During ten consecutive weeks, GPs in the intervention group received evidence-based online feedback on the management of suspected RTIs. In patients with acute bronchitis, the intervention reduced the antibiotic prescribing rate from 71.6% to 56% (control group from 61.2% to 52%). In patients with acute otitis media, the intervention reduced the antibiotic prescribing from 94.8% to 86.2% (no change in the control group). In all RTIs, the intervention reduced antibiotic prescribing rate from 37.4% to 28.1% (control group from 29% to 27.2%). Online evidence-based feedback is effective for reducing antibiotic prescribing in patients with RTIs attending primary care in South America.

## 1. Introduction

Inappropriate use of antibiotics generates Antimicrobial Resistance (AMR), which represents a serious threat for societal development due to its health and economic impact [1,2].

Antimicrobial resistance (AMR) is an increasing global problem. Studies have found a high prevalence of AMR in several countries in Latin America, particularly for pathogens involved in community acquired respiratory tract infections (RTIs) [3,4,5] such as *Streptococcus pneumoniae*, *Haemophilus influenza*, and *Moraxella catarrhalis*. For example, the latest report from the World Health Organization on antimicrobial resistance found a prevalence of *Streptococcus pneumoniae* resistant to penicillin of 65% in Bolivia and 30% in Argentina [1].

There is a lack of data on the use of antibiotics in South America. In a previous study [6], we found that general practitioners (GPs) from Argentina prescribed antibiotics on average to 41% of the patients consulting with respiratory tract symptoms. Population-based data have shown an increase in antibiotic consumption [7]. This study analyzed consumption of antibiotics between 1997 and 2007 in eight Latin American countries. In general, there was an increase in consumption of antibiotics and great variation across countries. For example, in Uruguay, during this period, the consumption of antibiotics increased from 5.43 Defined Daily Dosis per 1000 inhabitants per day (DID) in 1997 to 8.90 DID in 2007. Argentina maintained high levels of consumption 16.64 DID. In all countries, there was a significant increase in the consumption of broad-spectrum antibiotics. These types of antibiotics are those with the highest probability of triggering AMR. Due to the high prevalence of AMR and high use of antibiotics in South America, effective interventions should be implemented to reduce antibiotic overprescription.

The implementation of effective interventions to reduce inappropriate prescription of antibiotics in primary care in Latin America is challenging. Not only is it important to take into consideration the fragmented health care systems (e.g., differing populations between general practitioners depending on the type of health insurance the patient belong to), but also the problem of poor compliance with regulations to prohibit the sale of antibiotics over the counter [8].

RTIs are the most common reasons for antibiotic prescribing in primary care [9]. Most RTIs are caused by a virus, and in the majority of patients, antibiotics have no beneficial effect [10,11]. In a previous observational study [6], high antibiotic prescribing rates for acute bronchitis and otitis media were found. Acute bronchitis is mainly a viral infection [11], while the prescription of antibiotics in patients with Acute Otitis media requires the fulfillment of specific criteria [10].

Several strategies have been developed to reduce inappropriate prescribing of antibiotics in primary care. A systematic review comparing different interventions in primary care found that interventions aimed at reducing overall antibiotic prescribing were more effective than interventions focusing on the right choice of antibiotics [12]. A more recent review concluded that antibiotic use could be improved by educational interventions such as dissemination of printed/audiovisual educational materials, group education, personal or group feed-back, individual outreach visits, reminders at the time of prescribing, computer-assisted decision-making systems, among others. Both reviews agree on pointing out that a greater effect is achieved with multi-faceted interventions [12,13].

It is difficult to assess which element of a multifaceted program is the one driving behaviour change. Hence, as part of the quality improvement program: HAPPY AUDIT (Health Alliance for Prudent Prescribing, Yield and Use of Anti-microbial Drugs in the Treatment of Respiratory Tract Infections), we sought to assess the added effect of online evidence-based feedback.

HAPPY AUDIT South America was launched in 2013. GPs from Argentina, Bolivia, Uruguay, and Paraguay were invited to participate in a quality improvement cycle to decrease the inappropriate prescription of antibiotics in patients with suspected RTI. As part of the quality improvement cycle, all GPs collected data about their prescribing decision between June–August 2014. In March 2015, GPs in every country were invited to a two-day meeting to talk about the personal prescribing report in comparison to the general report at country and the South American level. Furthermore, GPs discussed about the challenges for the diagnosis process, as there is no national guidelines and availability of point-of-care-tests (POCTs). Afterwards, they were given educational material for their patients about the most common respiratory tract infections. Between June–August 2015 GPs registered again their prescribing decisions. During this second data collection, some GPs were randomly exposed to the evidence-based online feedback intervention.

This analysis aimed at assessing the effectiveness of online evidence-based feedback on reducing antibiotic prescribing in patients with suspected respiratory tract infection, especially in patients with the diagnoses of acute bronchitis and acute otitis media in four South-American countries.

## 2. Results

**Figure 1:**
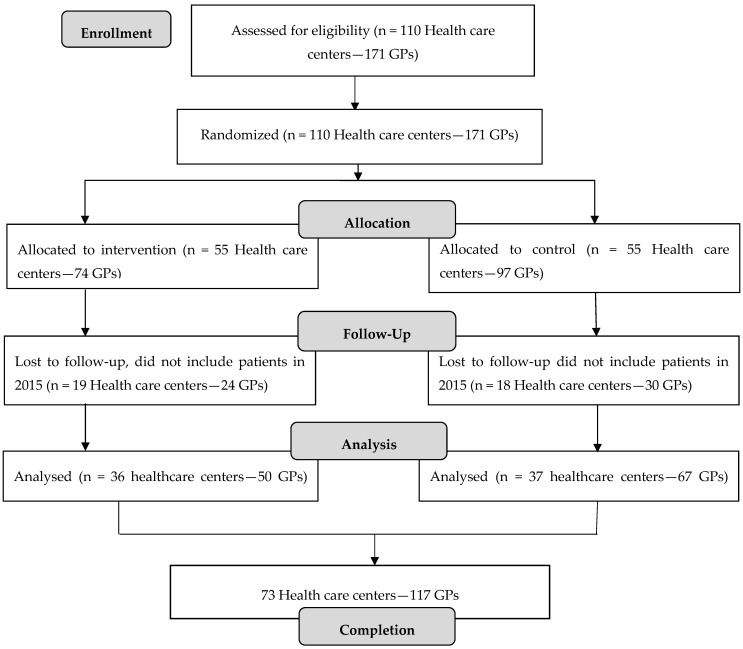
Flow chart of the study population.

Table 1 shows baseline characteristics of participating GPs. There were no statistically significant differences in baseline characteristics between GPs in the intervention and control group. In 2014, 110 health care centres were randomized to intervention or control group. There were completed data from 73 health care centres; 36 (50 GPs) in the intervention arm and 37 (67 GPs) in the control arm—see Figure 1.

In 2014 (before intervention), 8482 patients were registered. 2805 (33%) patients received an antibiotic prescription. In 2015 (after intervention), 8052 patients were registered; 2225 (28%) received an antibiotic prescription.

Adherence and use of the online feedback intervention were tracked in the program surveyexact. Participation was above 90% for each of the clinical cases.

Table 2 shows the antibiotic prescribing rates for acute bronchitis and acute otitis media before and after the intervention. For acute bronchitis, the intervention group reduced the antibiotic prescribing rate from 71.6% to 56% (difference 15.6%, 95%Confidence Interval (CI) 8.3; 22.7), and the control group reduced the antibiotic prescribing rate from 61.2% to 52.1% (difference 9.1%, 95%CI 2; 16). For acute otitis media, the intervention group reduced the antibiotic prescribing rate from 94.8% to 86.2% (difference 8.6%, 95%CI 0.5; 18). There was no change in antibiotic prescribing in the control group. For all RTIs, the intervention group reduced the antibiotic prescribing rate from 37.4% to 28.1% (difference 9.3%, 95%CI 7.1; 11), and the control group reduced the antibiotic prescribing rate from 29% to 27.2% (difference 1.8%, 95%CI 0.08; 3.6).

Table 3 shows the results of the hierarchical logistic models. There was a significant reduction in antibiotic prescribing for acute bronchitis in both the intervention and the control group. The reduction in antibiotic prescribing in patients with acute bronchitis was higher in the intervention group (OR 0.25 95%CI 0.15; 0.42) than in the control group (OR 0.60 95%CI 0.38; 0.94), (*p* = 0.001).

## 3. Discussion

### 3.1. Summary of Main Findings

Overall, there was a decrease in antibiotic prescribing for patients with acute bronchitis, acute otitis media and in all patients with a suspected RTI. The reduction was significantly higher in the intervention group compared to the control group. A possible explanation for the reduction in antibiotic prescribing in both groups can be the participation in the HAPPY AUDIT cycle alongside the cluster randomized control trial. This could have made all participants more aware of their antibiotic prescribing. Nonetheless, a larger effect in the intervention group indicates that exposure to online feedback on evidence-based management of RTI can bring about a larger effect in the reduction of unnecessary prescriptions.

### 3.2. Strengths and Weaknesses of the Study

The results are based on data from 117 GPs and 16,535 patients across four countries in South America. The large sample size allowed accurate assessment of the decrease in antibiotic prescribing for the selected diagnoses: acute bronchitis, acute otitis media, and overall.

The online feedback intervention was assessed as an added feature of the quality improvement program HAPPY AUDIT. This program is based on the methodology of a medical audit developed by the Audit Project Odense group [14]. This methodology relies on voluntary participation and a bottom-up approach in which GPs themselves set their own improvement goals.

On the one hand, the voluntary participation of GPs affects the external validity of these findings. Previous research has shown that GPs participating in quality improvement programs or research tend to prescribe fewer antibiotics [15,16]. Part of the reduction may have been caused by the desire to improve their prescribing behaviour. Nonetheless, the intervention group had a larger decrease in comparison to the control group (also motivated to decrease their prescribing).

On the other hand, the voluntary participation guarantee that the recording of the data did not suffer from observation bias (i.e., change in prescribing behaviour due to participation in a research program). All participants were interested in knowing their prescribing pattern to set their own quality improvement goals. Hence, they were very interested in recording data as requested to obtain an accurate assessment of the change in their prescribing pattern. Regarding the second registration, GPs were not informed that the primary success outcome for the assessment of the online feedback intervention was a decrease in prescriptions for acute bronchitis. This ensured that they were not focusing on this diagnosis or changing the label from acute bronchitis to Pneumonia to justify the prescription of antibiotics (see Supplementary Materials Table S1).

There were no differences in the exposure to the intervention among the participating practices. All practices in the intervention group received the same information through the same channel (e-mail/surveyxact program). This is an important characteristic of the intervention because it overcomes the limitations of different peer academic detailers, who might influence the results of the intervention depending on their pedagogical skills [17].

Allocation bias at the practice level was reduced by using a computer-based allocation process. The allocation was performed before the first data collection, so there was no information about the prescribing pattern of the GPs. Furthermore, the person running the allocation did not know the GPs or have any contact with the GPs during the whole study.

Cross-contamination was minimised by allocation at the practice level. This ensured that GPs working in the same health care center were in the same randomization groups. Furthermore, the allocation was stratified by solo (one GP per health care center) or group (two or more GPs per health care center) practices. It was done to achieve a balance in the number of GPs in both randomization groups. Due to the large variation in the number of GPs per health care centre, there were more GPs in the control group.

We sought to reduce diagnostic misclassification by using the same data collection instruments before and after the intervention. The data collection instrument was designed based on the APO methodology [18], so GPs used a very small amount of time during the consultation to record the main characteristics of the patient and the treatment decision.

The cluster-randomized trial was carried out alongside the HAPPY AUDIT quality improvement program. This methodology relies on data collection under daily practice conditions. Hence, we cannot rule out diagnostic misclassification. There are no national guidelines, and the lack of POCTs makes it difficult to standardized diagnostic criteria in everyday practice. However, the similar distribution of diagnoses between 2014 and 2015 may indicate that there was no a differential misclassification during the two periods of data collection (Supplementary Materials Table S1).

There was a one-year difference between the two data collection periods. During this period, GPs could have been exposed to other types of interventions like public health campaigns or courses about appropriate use of antibiotics, which would have contributed to the decrease in the prescription of antibiotics. Unfortunately, for the GPs working within the South American context, there is a worrisome lack of engagement by the public health authorities and other scientific bodies to decrease the unnecessary prescription of antibiotics in primary care. The only source of information about appropriate use of antibiotics the GPs were exposed to during 2014 and 2015 was the material provided by HAPPY AUDIT.

At baseline, there was a higher proportion of high prescribers in the intervention group (32%) in comparison to the control group (21%). It may explain the difference in the prescription of antibiotics between the intervention and control group. A hierarchical model to assess whether prescribing style was a confounder of the effect of the intervention between the two groups was tested. The strength and direction of the results did not change (Supplementary Materials Table S2).

Finally, prescribing data were only compared before and after the intervention without multiple follow-up data points; hence, we cannot completely rule out that part of the decrease may have been caused by the regression-to-the-mean effect [19].

### 3.3. Comparison with Other Similar Studies

There are very few studies assessing the effectiveness of online feedback interventions on physicians’ performance. Generally, online feedback programs target patients to change their lifestyle. One study [20] performed in Canada aiming to increase awareness and use of evidence-based research in clinical practice and to increase use of Internet-based resources for continuing medical education concluded that on-line case-based discussion is a promising strategy for encouraging family physicians to access current research. This study provides just a general conclusion that online feedback may be effective, but it does not assess the effects within a specific area.

There is a lack of studies on evidence-based feedback interventions aimed at decreasing inappropriate prescribing of antibiotics in the South American context. A small study performed in a hospital in Bogota-Colombia used online learning targeting general practitioners and reduced the prescribing rate of antibiotics in patients with suspected RTI [21]. Nonetheless, the results cannot be extrapolated to primary care due to different working conditions and different patient populations.

A study performed in the USA [22] targeting antibiotic prescribing for non-complicated acute bronchitis in adults also showed a substantial decline in antibiotic prescribing rates in the intervention group (from 74% to 48%; *p* < 0.003) and not in the control group (78% to 76%; *p* = 0.81). The reduction in prescribing rates in this study was larger than in our study. A plausible explanation for that difference is that their intervention targeted specifically acute bronchitis while the intervention in our study targeted all patients with suspected RTIs and GPs did not know that the primary outcome was a reduction in the proportion of prescriptions in patients with suspected acute bronchitis.

A multinational study [23] performed in Europe assessed the effects of internet-based training on antibiotic prescribing for acute respiratory tract infections. The European study included four arms: usual care; internet-based training to use a point-of-care CRP test; internet-based training in enhanced communication skills; or combined training in CRP testing and enhanced communication skills. Similar to our study, they focused on interactive interventions rather than just providing educational information. In line with our study, there was a reduction in prescription of antibiotics. Interactive methods are better than those that present information without requiring feedback from the recipient [24]. The larger effect of their intervention in comparison to our intervention can be explained by the following differences in the content of the intervention. First, our intervention did not include the use of any POCT. The use of the CRP test has demonstrated to be effective without additional interventions for decreasing prescription of antibiotics as it helps the GP to rule out a bacterial infection and helps the GP to establish a dialogue with the patient about the need for antibiotics [25,26]. Second, Little et al. intervention required more “study time” for each of the modules. Our intervention sought to use very little time from the GP to encourage them to read the clinical case, answer back and read the key literature using maximum one hour per week.

### 3.4. Relevance of the Findings

There are very few studies assessing the effectiveness of interventions to improve antibiotic use performed in Latin American countries [12,13]. Hence, more trials testing the same intervention or comparing online feedback with use of diagnostic test are required to get a robust assessment of the effect of these interventions within the Latin American context.

Our study proved that an online-based interactive intervention could reduce the prescribing of antibiotic for RTIs among GPs in the Latin American context, but we do not know if that leads to a reduction in antibiotic use or on the contrary leads to a higher use of antibiotic or other medications without prescriptions [8]. This can be particularly important in the South American context due to the non-negligible amount of antibiotic sales without prescription. Further research should focus on this problem and future studies should include inter-sectoral interventions.

Finally, Internet training has proved to be helpful and has the advantage that it can be disseminated widely at low cost and does not require highly trained outreach facilitators to be on site, which is especially important in low-income countries and rural areas.

## 4. Materials and Methods

### 4.1. Design

A cluster randomized two-arm controlled trial was carried out alongside the quality improvement program HAPPY AUDIT (Health Alliance for Prudent use and yield of antibiotics in patients with suspected RTI). Health care centres were the unit of allocation and intervention. Individual data at patient and GP level were collected and analysed.

### 4.2. Setting and Participants

GPs from the medical associations in Bolivia, Argentina, Paraguay, and Uruguay were invited to participate in the quality improvement program HAPPY AUDIT. All GPs who voluntarily accepted toparticipate in HAPPY AUDIT were randomized for participation in the cluster randomized trial (Figure 1).

### 4.3. Ethics

Ethics approval was granted in each country by the following authorities. Bioethics Committee, Posadas, Misiones—Argentina (File No. 022014). Department of Quality, Education and Research at “Caja Nacional de Salud” La Paz—Bolivia (File No. 29/05/2014) and the Ethics Committee of “Arco Iris” Hospital. Ministry of Health and Welfare, Seventh Health Zone, Encarnación—Paraguay (File No 116/2014). Ethics Committee for research projects at the Faculty of Medicine, University of the Republic, Montevideo—Uruguay (File No. 070153-000309-14).

### 4.4. Sample Size Calculation

Power calculation was based on the results of an earlier study of RTIs in Argentina [6]. According to HAPPY AUDIT, the antibiotic prescribing rate for acute bronchitis was about 60%. Thus, in order to demonstrate a 20% reduction of prescribing rate, with a power of 80%, a statistical significance level of 5% and an intra-class correlation of 0.02, we estimated to include 110 practices, 55 in the intervention group and 55 in the control group, and each practice should include at least 40 patients.

### 4.5. Data Collection and Outcomes

In each country, GPs registered patients with suspected RTI according to the HAPPY AUDIT procedures. It means, before the first data collection, all the GPs attended a course about the use of the data collection form and diagnosis of the most common respiratory tract infections. In South America, there is not access to point-of-care tests (POCTs), and there are no national guidelines about the diagnosis and management of RTIs. The diagnoses were only based on clinical information. For example, acute bronchitis was diagnosed on clinical basis, as C-reactive protein is not available. The secondary outcomes were: (a) reduction in prescription of antibiotics in patients with acute otitis media; (b) overall reduction in prescription of antibiotics in patients with suspected RTI. GPs registered the following information in a standardized form: age, sex, symptoms, signs, anticipated focus of infection, suspected etiology, and treatment (antibiotic prescribing).

### 4.6. Random Assignment

To avoid cross-contamination, the randomization procedure was done at the practice level. Practices were stratified solo practices (only one GP per health care centre) or group practices (two or more GPs per health care centre), and for each strata, the intervention was randomly assigned with half of practices in the intervention group and half in the control group. The person in charge of running the computer-based random assignment did not know the participants and had not information about their prescribing pattern.

### 4.7. Intervention

All GPs participating in the HAPPY AUDIT quality cycle were randomized either to intervention (evidence-based online feedback) or control (no exposure to the evidence-based online feedback). GPs in the intervention group received an e-mail with a link to an on-line intervention program that included the following three modules:

(a) Presentation of a clinical case: a patient with a RTI;
(b) Multiple choice questions focusing on evidence-based decision rules and treatment proposals (three questions per clinical case);
(c) Overall feedback with correct answers and references to key literature.

In total, GPs received ten clinical cases with questions during June–August 2015. GPs had one week to send the answer back. After one week, the GPs received the right answer from the previous clinical case with key literature and a new clinical case.

The online feedback was sent through the surveyexact program. The program registered the number of respondents per clinical case.

### 4.8. Statistical Analysis

Two hierarchical logistic regression models were developed for each of the following outcomes: (a) prescription of antibiotics in patients with acute bronchitis; (b) prescription of antibiotics in patients with acute otitis media and (c) prescription of antibiotics in all patients with suspected RTI. The first model tested differences in antibiotic prescribing rates before-after intervention for each group (intervention and control). The second model tested the added effect of the evidence-based online intervention. It tested the interaction between randomization group and year. The structure of the data was maintained by including two random intercepts: (a) One at the practice level and; (b) one at the GP level. All analyses were performed in the R programming language and environment v3.3.2 using the lme4 and nnet package [27].

## 5. Conclusions

Online evidence-based feedback is effective for reducing antibiotic prescribing in patients with suspected respiratory tract infection attending primary care in South America and it is a tool that can be widely disseminated at low cost without requiring highly trained facilitators, which is especially important in low-income countries and rural areas.

## Figures and Tables

**Table 1:** Baseline characteristics of participating general practitioners (GPs).

Characteristics	Intervention Group (36 Groups; 50 GPs)	Control Group (37 Groups; 67 GPs)	*p*
Women	33 (66%)	45 (67%)	0.8
Age *	40 (8)	38 (8)	0.1
Specialization in general practice	28 (56%)	35 (52%)	0.6
>10 years work experience	17 (34%)	24 (36%)	0.8
Urban practice	29 (58%)	37 (55%)	0.7
Number of consultations per day *	24 (8)	21 (11)	0.1
High prescribers ^¥^	16 (32%)	14 (21%)	0.1

* Mean (SD), ^¥^ GPs prescribing antibiotics to more than 75% of their patients.

**Table 2:** Prescription of antibiotics in 2014 and 2015.

Outcomes	2014		2015		Difference in Proportions
	Patients	Prescribed Antibiotics (%)	Patients	Prescribed Antibiotics (%)	
Acute bronchitis					
Intervention	381	71.6	327	56	15.6 (CI 8.3; 22.7)
Control	431	61.2	378	52.1	9.1 (CI 2; 16)
Total	812	66	705	54	12 (CI 6.9; 16)
Otitis media					
Intervention	155	94.8	87	86.2	8.6 (CI 0.5; 18)
Control	138	79	134	82	3 (CI −6; 12)
Total	293	87	221	83.7	3 (CI −3; 9.9)
All RTI					
Intervention	4050	37.4	3644	28.1	9.3 (CI 7.1; 11)
Control	4433	29	4408	27.2	1.8 (CI −0.08; 3.6)
Total	8483	33	8052	28	5 (CI 3.9; 6.8)

**Table 3:** Reduction in prescription of antibiotics in patients with suspected RTI within and across randomization groups.

Outcomes	OR	95%CI	*p* Value ^¥^
Acute bronchitis			
Intervention	0.25	0.15; 0.42	0.001
Control	0.60	0.38; 0.94	
Acute Otitis			
Intervention	0.32	0.10; 1.01	0.05
Control	1.06	0.49; 2.28	
All RTI			
Intervention	0.59	0.53; 0.65	<0.001
Control	0.82	0.74; 0.91	

^¥^ *p* value of the interaction term: intervention * year—added value of the intervention.

## Supplementary Materials

The following are available online at www.mdpi.com/2079-6382/6/4/38/s1, Table S1: Distribution of diagnosis before and after the intervention; Table S2: Reduction in prescription of antibiotics in patients with suspected RTI within and across randomization groups—Adjusted by prescribing style.
