# Supplementary material for: Decreasing Inappropriate Use of Antibiotics in Primary Care in Four Countries in South America—Cluster Randomized Controlled Trial
Source: Antibiotics (Basel). 2017 Dec 14;6(4):38. doi: 10.3390/antibiotics6040038 (PMC5745481; doi:10.3390/antibiotics6040038)
Supplement: Supplementary file 1 [file antibiotics-06-00038-s001.pdf]

Supplementary Materials

Supplementary Tables

Table S1. Distribution of diagnosis before and after the intervention

Diagnoses	2014 (n = 8483) Missing = 72 (0.8%)	2015 (n = 8052) Missing = 5 (0.06%)
Common cold	3358 (39.5%)	3394 (42.1%)
Acute Otitis Media	293 (3.4%)	221 (2.7%)
Acute sinusitis	199 (2.3%)	174 (2.1%)
Acute pharyngitis	1471 (17.3%)	1421 (17.6%)
Acute tonsillitis	944 (11.1%)	889 (11%)
Influenza	812 (9.5%)	705 (8.7%)
Acute bronchitis	269 (3.1%)	307 (3.8%)
Pneumonia	141 (1.6%)	156 (1.9%)
COPD	213 (2.5%)	241 (2.9%)
Other RTI	711 (8.3%)	539 (6.6%)

Table S2. Reduction in prescription of antibiotics in patients with suspected RTI within and across randomization groups—Adjusted by prescribing style

Outcomes	OR	95%CI	*p* Value[¥]
Acute bronchitis			
Intervention	0.24	0.15;0.40	0.001
Control	0.62	0.41;0.95	
Acute Otitis			
Intervention	0.37	0.12;1.11	0.05
Control	1.3	0.4;2	
All RTI			
Intervention	0.60	0.54; 0.66	<0.001
Control	0.83	0.75;0.92	

[¥] *p* value of the interaction term intervention*year. Prescribing style = binary variable divided into high and low prescribers. High prescribers are those GPs prescribing antibiotics to more than 75% of their patients.
